# Autophagy in reproduction and pregnancy-associated diseases

**DOI:** 10.1016/j.isci.2024.111268

**Published:** 2024-10-28

**Authors:** Asmita Singh, Maira L. Perez, Oleksandr Kirsanov, Elizabeth Padilla-Banks, Carlos M. Guardia

**Affiliations:** 1 Placental Cell Biology Group, National Institute of Environmental Health Sciences, National Institutes of Health, Research Triangle Park, Durham, NC, USA

**Keywords:** physiology, pathophysiology, molecular biology, cell biology

## Abstract

As advantageous as sexual reproduction is during progeny generation, it is also an expensive and treacherous reproductive strategy. The viviparous eukaryote has evolved to survive stress before, during, and after pregnancy. An important and conserved intracellular pathway for the control of metabolic stress is autophagy. The autophagy process occurs in multiple stages through the coordinated action of autophagy-related genes. This review summarizes the evidence that autophagy is an integral component of reproduction. Additionally, we discuss emerging *in vitro* techniques that will enable cellular and molecular studies of autophagy and its associated pathways in reproduction. Finally, we discuss the role of autophagy in the pathogenesis and progression of several pregnancy-related disorders such as preterm birth, preeclampsia, and intra-uterine growth restriction, and its potential as a therapeutic target.

## Graphical abstract

**Figure:**
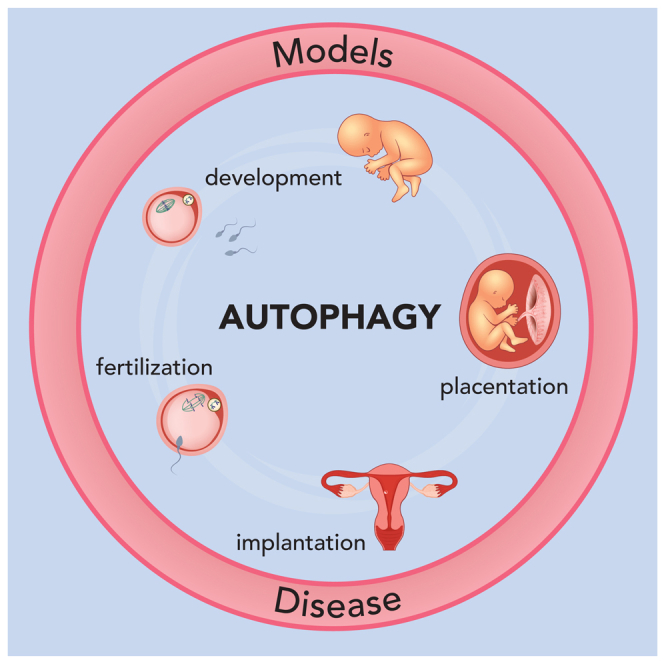

## Introduction

Sexual reproduction leads to genetic material exchange during progeny generation.^1^ Shuffling and rearranging parental genomes provide, in most effective cases, enough genetic diversity in the offspring to adapt to and thrive in new environments,^2^ even though theoretical models cannot fully support some of these benefits.^3^^,^^4^ This apparent evolutionary advantage over asexual reproduction imposes many costly challenges. It requires mating, increased time and energy, and is intrinsically and molecularly more complex. The development of gonads that produce competent gametes, the physical need of such gametes to encounter the correct spatiotemporal conditions, and the proper generation of a new organism are all extremely critical steps of sexual reproduction.^5^ In particular, viviparous eukaryotes face extra challenges in comparison to those with external organismal development; the former requires the evolution of pregnancy-specific mechanisms of protection, nurturing, survival, and delivery.^6^^,^^7^ Thus, it is not surprising that the rate of successful pregnancies is low in some species, and that survival mechanisms in response to stress (metabolic, infection, and so forth) have evolved with pregnancy to allow eukaryotic cells to cope with different assaults before, during, and after pregnancy.^8^^,^^9^

**Figure 1:**
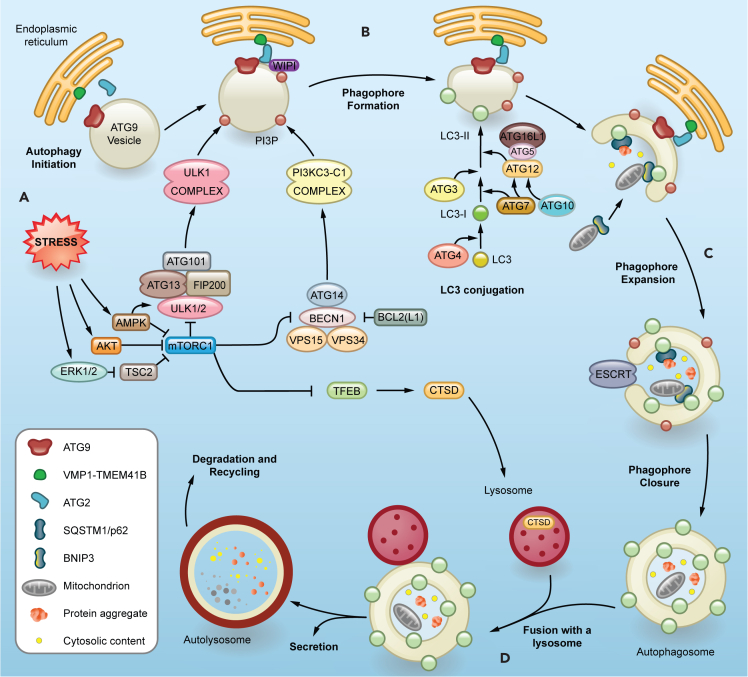
Molecular players in the autophagy pathway Schematic overview of the mechanism of autophagy. (A) Cellular stress results in the formation of pre-autophagosomal structures. Autophagy initiation involves the mTOR inhibition and activation of the ULK complex, which recruits class III phosphatidylinositol 3-kinase complex C1 (PI3KC3-C1) to the phagophore formation site. This lipid kinase complex is responsible for phosphatidylinositol 3-phosphate (PI3P) generation on ATG9 vesicles, the now speculated membranous seed for phagophore formation. WIPI proteins bind to PI3P and subsequently recruit ATG2, which controls phospholipid trafficking into the growing phagophore from other membrane sources, mainly the endoplasmic reticulum. (B) The growing phagophore membrane is decorated with the ATG8/LC3 family of proteins and cargo receptors by a ubiquitin-conjugation-like machinery. LC3 is first processed from its precursor form to LC3-I through cleavage by ATG4, exposing a C-terminal glycine. LC3-I is then lipidated with phosphatidylethanolamine to form LC3-II, which integrates into autophagosomal membranes. (C) Sequestration of selectively ubiquitin-tagged or non-selective cargo recruitment into the expanding phagophore. This is followed by the sealing of the phagophore membrane by ESCRT machinery. (D) A fully formed double membrane autophagosome fuses with a lysosome to assist in the degradation, recycling, and secretion of all the encapsulated material.

One highly conserved and ubiquitous mechanism of cellular and organismal survival is autophagy. The autophagic process involves the engulfment of intracellular materials in double-membraned autophagosomes that fuse with lysosomes for degradation (previously reviewed^10^^,^^11^^,^^12^). Autophagy proceeds over several stages with the coordination of core autophagy-related (ATG) genes and key accessory genes to perform essential functions (Figure 1). The initiation of autophagy requires the activation (AMPK, ULK1) and inhibition (AKT, mTOR) of several kinases involved in transducing intra- and extracellular signals of stress^13^^,^^14^ (Figure 1A). Some examples of these stressors are low levels of free amino acids, glucose, and oxygen, the presence of exogenous pathogens, misfolded proteins, and damaged organelles. In response to these stressors, the ULK1 complex (FIP200, ULK1 or ULK2, ATG13, and ATG101) is recruited to a pre-autophagosomal structure (PAS) rich in phosphatidylinositol 3-phosphate (PI3P) that forms in close vicinity to an "omegasome," a subdomain of the endoplasmic reticulum (ER).^15^^,^^16^^,^^17^ The main autophagy proteins involved in PI3P generation on the PAS are part of the class III phosphatidylinositol 3-kinase (PI3K) complex I (PI3KC3-C1) composed of BECN1, PIK3R4/VPS15, PIK3C3/VPS34, and ATG14.^18^^,^^19^ Next, WD-repeat protein interacting with phosphoinositides (WIPI) 1–4 proteins bind to the PI3P in the PAS and recruit the next two important components of phagophore expansion: ATG2 proteins and the ATG16L1 complex.^20^^,^^21^ ATG2A and ATG2B are cytosolic lipid transfer proteins that transport phospholipids to the PAS and growing autophagosome.^22^ They work together with a set of scramblases to keep membrane integrity at the transfer sites^23^ (Figure 1A). VMP1 and TMEM41B function as scramblases in the ER,^24^ while ATG9 proteins fulfill the same function in small Golgi-derived vesicles.^25^^,^^26^^,^^27^ Recent discoveries suggest that ATG9-containing vesicles are the actual PAS where PI3P and its effectors are recruited^28^^,^^29^^,^^30^ (Figure 1A). In addition, other ER subdomains and membranous organelles often surround nascent phagophores, likely contributing to their growth and maturation, though how the cell coordinates the different roles of these components is still an area of intense research.^31^^,^^32^^,^^33^^,^^34^^,^^35^^,^^36^^,^^37^ While the phagophore membrane expands, a ubiquitin-conjugation-like machinery (ATG16L1 complex) decorates it with the ATG8/LC3-family proteins and cargo receptors^38^ (Figure 1B). ATG7 (E1), ATG3 and ATG10 (E2), and ATG5-ATG12-ATG16L1 (E3) coordinate the covalent conjugation between the C-terminus of ATG8/LC3 (ubiquitin-like) and the amino group of phophatidylethanolamine enriched in the phagopore by the assistance of ATG4. The lipidation of ATG8 is required for an efficient phagophore formation, cargo recruitment, and downstream fusion with lysosomes.^39^ The rapid expansion of the phagophore can engulf mostly cytosolic content nonspecifically.^40^ Perhaps more importantly, phagophore generation and growth can also occur around and specifically recruit cytoplasmic content tagged for degradation during selective autophagy^41^ (Figure 1C). This is mediated by cargo receptors, such as sequestosome 1 (SQSTM1/p62), that recognize both cargo and autophagosome components.^42^ Each one of these selective autophagy pathways requires specific cargo receptors that contain an LC3-interaction region (LIR) motif. The canonical LIR sequence follows the [W/F/Y]-X-X-[L/I/V] pattern (X represents any amino acid preferably small and flexible). Most of the autophagy cargo receptors can oligomerize^43^ in order to recruit cargo to the phagophore through direct interaction with ubiquitin and lipidated LC3 proteins. Examples of selective autophagy are mitophagy, ER-phagy, lysophagy, and lipophagy, which represent the specific degradation of mitochondria, ER, lysosomes, and lipid droplets, respectively, among others.^44^ In case of selective elimination of damaged mitochondria, for example, the ubiquitin kinase PTEN induced kinase 1 (PINK1) recruits, stabilizes, and phosphorylates Parkin (PRKN), an E3 ubiquitin protein ligase, which in turn ubiquitinates several outer membrane mitochondrial proteins. These ubiquitin tags are recognized by LIR domain-containing autophagy receptors, which direct the selective mitochondria targeting to autophagosomes.^45^ BNIP3 and BNIP3L/NIX are well-known selective mitophagy receptors for the delivery of damaged mitochondria to autophagosomes.^46^^,^^47^ Less studied are the processes that lead to phagophore closure, which generates a mature autophagosome,^48^^,^^49^ but a clear role of the endosomal sorting complexes required for transport (ESCRT) machinery has been established.^50^ Once fully formed, the autophagosomes are capable of fusing to late endosomes and lysosomes to assist with the degradation of the encapsulated material,^51^ their secretion,^52^ and recycling^53^ (Figure 1D). This allows the cell to control the stressful insult that prompts the autophagic response.

In this review, we summarize the essential role of the autophagy machinery at different stages of a healthy pregnancy (Table 1) while exploring the central concept that autophagy serves as a surveillance mechanism that secures positive reproductive outcomes by monitoring resource availability during cellular stress. We explore the evidence and gaps in knowledge for the role of autophagy in the formation of competent gametes, fertilization and implantation, the development of the placenta, and parturition. The role of autophagy in mammalian embryonic development and organogenesis has been extensively reviewed and readers are directed to these articles for more information.^54^^,^^55^ Additionally, we provide an overview of the latest tools that can be used to interrogate the function of autophagy during early stages of human development *in vitro*. Finally, we review the role of autophagy in pathogenesis and disease progression for a myriad of pregnancy-related disorders. With a focus on murine and human models, this review highlights the most fundamental and recent findings in the field.

**Table 1:** A summary of important studies on autophagy in pregnancy

Stage	Study with the main phenotype	Citation
Sperm development	• Reduced levels of conjugated LC3A/B and accumulation of SQSTM1/p62 in male germ cells lacking Atg5 • Abnormal sperm morphology from Atg7 conditional KO in male germ cells	Ref.^56^^,^^57^
	• An *Atg7* null mouse had impaired sperm motility and abnormal sperm morphology	Ref.^58^
	• Loss of *Atg5* and *Atg7* in Leydig cells declined sexual behavior in mice	Ref.^59^
Oocyte development	• *Becn1*^*+/−*^ (murine ovaries) showed a 56% reduction of germ cells on postnatal day 1 • *Atg7* deficient ovaries showed a total loss of germ cells	Ref.^60^
	• *Atg7* KO in germ cells had more than 50% oocyte cell loss during neonatal transition period	Ref.^61^
	• ATG5 and BECN1 KD in GCs resulted in decreased expression of genes associated with differentiation	Ref.^62^
	• *Becn1*-deficient mouse had a defect in progesterone production and preterm labor phenotype • Luteal cells lacking *Becn1* showed defective autophagy and steroidogenesis	Ref.^63^
Embryo implantation	• Sperm fertilization of *Atg5* null oocytes resulted in developmental failure beyond the 8-cell stage	Ref.^64^
	• Inhibition of mTORC1 pathways delays blastocyst development	Ref.^65^^,^^66^
Fetus and Placenta development	• *Rb1cc1/FIP200* conditional KO in murine uterus and hESC, and conditional *Atg16L1* KO in female mice (reproductive tract) had reduced fertility	Ref.^67^^,^^68^
	• KD of *ATG7* and *ATG5* resulted in deficiencies in decidualization in hESC	Ref.^69^
	• *Becn1*-deficient uteri showed improper uterine development	Ref.^70^
	• Autophagy-deficient EVT-derived placental cell lines displayed failed invasion	Ref.^71^^,^^72^^,^^73^
	• Bafilomycin A1 suppressed CTB fusion in primary human trophoblasts	Ref.^74^
	• Activation of autophagy using rapamycin in a pregnant mouse module resulted in excessive syncytialization	Ref.^75^
	• BECN1 inhibition or overactivation in villous CTBs decreased syncytialization	Ref.^76^
	• Homozygous germline mutation of *Tfeb* in mice led to fetal death between E9.5 and E10.5	Ref.^77^
	• *In vitro* trophoblast cells cultured in a hypoxic environment showed decreased levels of TFEB, LAMP1, LAMP2, and CTSD, and impairment of autophagic flux	Ref.^78^
	• Placental explants deficient in *ATG16L1* displayed heightened susceptibility to infections caused by *E. coli*	Ref.^79^
	• Infection of human trophoblast cells and mouse placentas by ZIKV induces the activation of autophagy • *Atg16L1* gene deficient mouse models reduced ZIKV infection in both placentas and fetuses	Ref.^80^
	• Active ZIKV replication in human and non-human primate placenta-derived STBs showed a decrease in expression of *ATG5* and *SQSTM1,* and protein levels of LC3B and SQSTM1	Ref.^81^
	• *Becn1* KO in precursor follicular granulosa murine cells resulted in failure of decidualization and predisposition to preterm labor	Ref.^63^
	• Placenta specific *Atg7* KO mice had a reduction in fetal growth	Ref.^82^
	• STB-specific *Atg7* KO mice inhibited fetal growth	Ref.^83^
	• *ATG5* KD in HTR8/SVneo cells exposed to high glucose levels showed decreased apoptosis and increased invasion of cells	Ref.^84^
	• JEG-3 trophoblast cells exposed to external ceramides displayed heightened autophagy activation	Ref.^85^
	• Placenta-specific *Atg7* KO mouse showed an increase in SQSTM1 expression, lower levels of TFEB expression, and high protein aggregates	Ref.^78^^,^^82^
	• Inhibition of PKCβ enhances autophagic flux and angiogenic imbalance in mouse placentas	Ref.^86^
Birth	• In *Atg5* KO and *Rraga*^Q66L^ mutant mice, AMPK- and mTORC1-dependent autophagy in the neonate is triggered by the sudden interruption of nutrients supplied by the placenta	Ref.^87^^,^^88^
	• Conventional *Atg3, Atg5, Atg7, Atg9a, Atg12*, and *Atg16L1* null mice presented fetal growth restriction, decreased plasma amino acid levels, and pre-natal or neonatal lethality within one day of birth	Ref.^87^^,^^89^^,^^90^^,^^91^^,^^92^^,^^93^^,^^94^
	• The fetuses from *Becn1*, *Atg3*, *Atg5*, *Atg7*, *Atg9a*, and *Atg16L1* KO mice displayed an IUGR phenotype and died on day 1 after birth	Ref.^79^^,^^87^^,^^90^^,^^91^^,^^92^^,^^95^
	• Loss of placenta-specific mTOR function in a mouse model impaired metabolic health of the offspring	Ref.^96^^,^^97^^,^^98^
	• Rapamycin treatment improved absorption in SM mice models	Ref.^99^^,^^100^

## Gamete formation and hormone regulation

**Figure 2:**
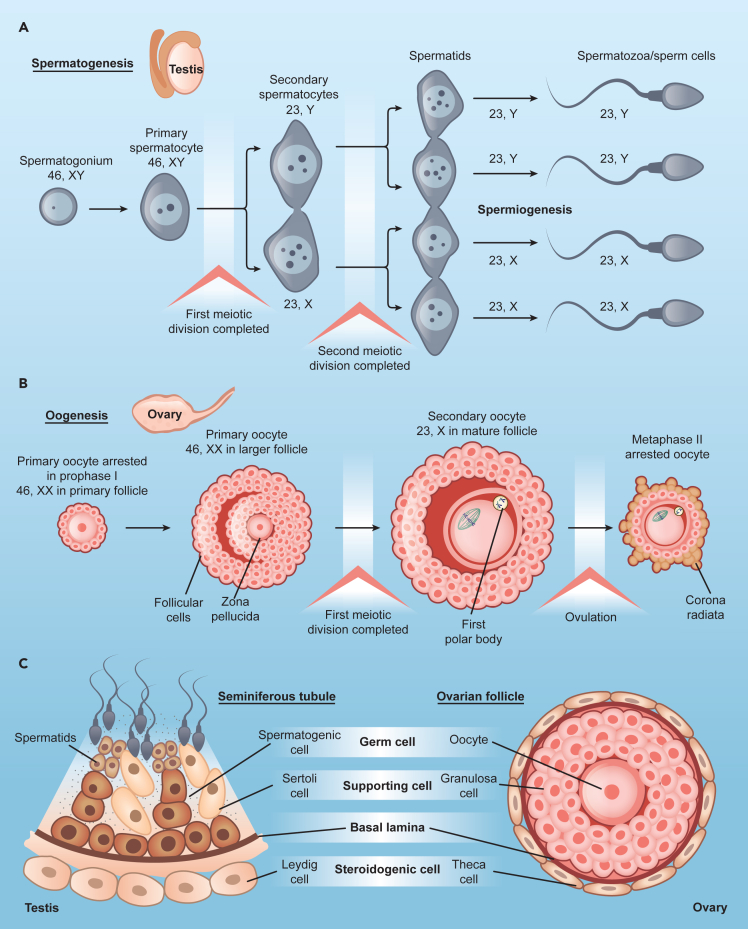
Schematic of gametogenesis The formation of an embryo after fertilization requires the proper development of a healthy egg and sperm in the process of gametogenesis. (A) The generation of sperm in males is known as spermatogenesis. The diploid primary spermatocytes arise from diploid spermatogonia which undergo two meiotic divisions to form haploid spermatozoa. First meiotic division produces two haploid secondary spermatocytes, each of which undergo second meiotic division to form two haploid spermatids. These spermatids acquire flagella to mature into spermatozoa. (B) In females, the formation of oocytes is known as oogenesis. The diploid primary oocyte undergoes two rounds of meiotic divisions to form a haploid secondary oocyte and a polar body. The primary oocyte is surrounded by follicular cells within the ovarian follicle. The follicle matures during the process of oogenesis to form a mature follicle which releases the egg during ovulation. (C) A comparison of seminiferous tubules in the testis and ovarian follicles highlights the different architecture and cell types surrounding the germ cells. The steroidogenic and supporting cells in each gonad are critical for the whole gametogenesis process and autophagy deficiency in these cells severely impacts the development of the gametes.

Central to sexual reproduction, gametogenesis (Figure 2) involves maintaining a balance between degradation and metabolic supply until gametes encounter fertilization. Deficient progenitor cells or with developmental disruptions do not produce viable or fertile gametes. In addition to potential problems during meiosis, each germ cell undergoes an enormous number of structural and functional changes to become a competent gamete. Not surprisingly, autophagy contributes to various aspects of gamete development and maturation.

In male germ cells, autophagy is essential for normal sperm development and function (Figure 2A). Murine male germ cells lacking *Atg5* showed reduced levels of conjugated LC3A/B and accumulated cargo receptor SQSTM1/p62.^56^ At a cellular level, these gametes had numerous, large residual bodies in different stages of spermatogenesis after their normal resorption within the seminiferous epithelium.^56^ Sperm had abnormal morphology, including disrupted head formation, abnormal acrosome and mid-piece formation, and abnormal mitochondria.^56^ This suggests a clear involvement of autophagy in spermiogenesis, the last and longest step of spermatogenesis (Figure 2A). Additionally, male germ cells lacking *Atg7* presented serious defects in acrosome biogenesis, resulting in round-headed sperm, which made the sperm appear globozoospermic.^57^ Interestingly, this conditional knockout (KO) only affected later stages of spermatogenesis, suggesting specific temporal roles of autophagy during sperm differentiation. Examination of *Atg7* null mouse showed the disruption of flagella biogenesis, with a reduction in sperm motility, retention of extra cytoplasm on the mature sperm head, and a disorganized cytoskeleton, probably due to failure of selective autophagy.^58^ This study demonstrated that autophagy in nurse and supporting cells ensures the correct development of the sperm (Figure 2C). In fact, *Atg5* and *Atg7* loss in Leydig cells affects sexual behavior in mice due to a decline in cholesterol trafficking and testosterone production, suppressing spermatogenesis.^59^ Additionally, using single-cell RNA sequencing analysis, transcriptional signatures of ATG genes were found to be dysregulated in sperm samples of infertile men presented with dysfunctional spermatogenesis.^101^ Therefore, autophagy is important for maintaining male fertility.

In the ovary, autophagy plays a role in the development of the primordial follicle pool, folliculogenesis, and progesterone synthesis (Figure 2B). At the time of birth, during the normal development of the primordial follicle pool in the mouse ovaries, a significant loss of the germ cell pool occurs.^102^^,^^103^ Autophagy protects against the excessive loss of oocytes during neonatal severe nutrient starvation which occurs just after birth and before feeding. Treatment with rapamycin (an mTOR inhibitor and thus, an autophagy enhancer) increases the number of surviving oocytes by 25–30% during mouse primordial follicle assembly.^104^ A high level of autophagy is thus associated with the suppression of apoptosis to prevent the loss of oocytes.^104^ Further, *Becn1*^+/−^ murine ovaries showed a 56% reduction of germ cells on postnatal day 1, and *Atg7*-deficient ovaries resulted in total loss of germ cells, demonstrating a role of autophagy in oogenesis.^60^ Germ cell-specific *Atg7* KO also showed more than 50% oocyte loss during the neonatal transition period.^61^ During folliculogenesis, only 1% of all follicles develop into mature oocytes, whereas other follicles undergo degeneration (atresia) at different developmental stages. Follicle atresia is thought to be triggered by massive granulosa cell (GC) apoptosis. Interestingly, autophagy is involved in the regulation of atresia and the development of the dominant follicle in different species, such as rats^105^^,^^106^ and humans.^107^ This was shown to be controlled in ovarian GCs by the mitochondrial voltage dependent anion channel 2 (VDAC2) protein which stabilizes the interaction between BECN-1 and BCL2L1 (an anti-apoptotic factor), leading to the inhibition of autophagy. This balance is a key switch for proper follicular development, offering potential strategies for enhancing female fertility by autophagy suppression.^108^ Interestingly, endolysosomal vesicular assemblies (ELVAs) in mouse oocytes were recently discovered.^109^ This unique structure accumulates autophagosomes, proteasomes, and protein aggregates that are only degraded at the final stages of oocyte growth and maturation. The dynamic of degradation and disassembly of these structures offers a potential explanation for how growing oocytes rely on autophagy only after maturation and fertilization (see next section).

The process of autophagy plays a critical role in regulating the synthesis of steroid hormones during gamete formation. Knockdown (KD) of *ATG5* and *BECN1* results in decreased expression of genes associated with the differentiation of ovarian GCs and those encoding steroidogenic enzymes hydroxy-delta-5-steroid dehydrogenase, 3 beta- and steroid delta-isomerase 1 (HSD3B1) and steroidogenic acute regulatory protein (STAR).^62^ This decrease is due to the accumulation of Wilms’ tumor 1 (WT1) transcription factor which contains an LIR motif and is degraded through autophagy. Furthermore, GCs from patients with biochemical premature ovarian insufficiency (bPOI) also show a reduction in the expression of key autophagy genes.^62^ Women with ovarian endometriosis exhibit an elevated ratio of serum progesterone/estradiol (P4/E2) and the P4-to-follicle index along with higher expression of P4 biosynthesis proteins.^110^ This increase is accompanied by a rise in BECN1 levels in GCs. The authors of this work suggested that in the case of ovarian endometriosis, increased activity of BECN1 results in increased hydrolyzation of low-density lipoproteins (LDL), cholesterol utilization, and lysosomal activity, resulting in elevated progesterone secretion.^110^ A similar study using a *Becn1*-deficient mouse also indicates *Becn1* is essential in lipid regulation and progesterone synthesis in luteal cells.^63^ Additionally, autophagy also degrades the negative regulators of cholesterol uptake pathways in Leydig cells, increasing cholesterol uptake for testosterone production in males.^59^ Finally, lipophagy is involved in gonadotropin-stimulated sex steroid synthesis in the human ovary (and testis).^111^ Hence, autophagy is essential to hormone-regulated successful gametogenesis by maintaining lipid supply for steroid synthesis, although the precise mechanisms remain unclear.

## Fertilization and preimplantation

The onset of fertilization triggers several critical processes, and autophagy is essential to their successful completion. Because autophagy has not been observed in unfertilized oocytes, the induction of autophagy is thought to be fertilization-dependent.^112^ Autophagy plays a role in providing nutrients and energy to the developing zygote and degrading paternal mitochondria to allow for maternal mitochondria transmission. The elimination of sperm-borne mitochondria during fertilization ensures normal preimplantation development.^113^ In *C. elegans,* paternal mitochondria are present in the embryo post-fertilization, which are marked by LGG-1/LGG-2 (ATG8-like proteins), signaling their autophagy degradation. Although these mitochondria are sequestered in double membrane vesicles indicating mitophagy, they are not ubiquitinated.^114^ In mammals, sperm mitochondria are ubiquitinated and tagged with several autophagosomal and lysosomal markers post-fertilization,^114^^,^^115^ which suggests the evolution of autophagy pathways to accommodate more complex fertilization events in mammals. Mice embryos expressing shRNA against *Sqstm1/p62, Tbc1d15, Pink1,* and mitochondrial E3 ligase *Mul1* exhibit suppression of paternal mitochondrial colocalization with LC3^114^^,^^116^^,^^117^ suggesting active mitophagy. Studies in porcine fertilization demonstrated that SQSTM1 participates in sperm mitophagy but not LC3, indicating the involvement of noncanonical ubiquitin-recognizing autophagy.^115^ Interestingly, a recent study using RFP-labelled mitochondria transgenic mice has found that the sperm mitochondria persisted until the morula stage and that autophagy did not participate in paternal mitochondria degradation post-fertilization,^118^ suggesting the involvement of other mechanisms besides autophagy.

Once the oocyte is fertilized, maternal mRNA and protein content are degraded in preparation for the new embryonic proteome. This process is part of what is known as the maternal-to-zygotic transition (MZT).^119^ Autophagy increases in the 1-cell stage embryo before a transient decrease and is then reactivated in the 2-cell stage embryo and maintained throughout the blastocyst stage.^64^^,^^65^^,^^112^ In mice, wild-type (WT) sperm fertilization of *Atg5* null oocytes was not affected, but *Atg5* null sperm fertilization caused failure during development beyond the 8-cell stage.^64^ This study indicates a critical role for autophagy in preimplantation embryogenesis and assistance in cytosolic remodeling during MZT stages. At the blastocyst stage, autophagy becomes particularly important for the development of the trophectoderm (the most external embryo layer). These cells produce 80% of the energy and amino acid turnover required for proper development and successful implantation of the blastocyst, while the inner cell mass remains quiescent.^120^

Temporary suspension of embryonic development, known as embryonic diapause, occurs prior to implantation and is conserved across mammals. As an evolutionary strategy, diapause ensures the survival of the embryo so optimal conditions of implantation and embryogenesis can be met.^121^ Studies have shown that autophagy is activated during diapause and that rapamycin reversibly delays blastocyst development.^65^^,^^66^ Despite this, researchers found that suppressing mTORC1 is not necessary nor sufficient to initiate this surge of autophagy.^122^ These results suggest that diapause-induced autophagy contributes to the maintenance of the blastocyst pluripotent state before implantation, but the mechanistic details of autophagy regulation at this developmental stage require future investigations.

Overall, autophagy facilitates the elimination of paternal mitochondria after fertilization, cytosolic remodeling during MTZ, and embryonic development, particularly at the blastocyst stage and embryonic diapause. As these dramatic cellular processes unfold, autophagy plays a crucial role in properly allocating resources and handling external challenges intracellularly until implantation and placentation occur.

## Implantation

Embryo attachment to the uterine lining is a critical factor for successful mammalian pregnancy. Autophagy plays a pivotal role in maintaining endometrial homeostasis. In the mouse, autophagy markers are increased in the endometrium at the time of implantation and rapidly decrease afterward.^123^^,^^124^ If autophagy is inhibited during pregnancy, the number of implantation sites are reduced.^124^ Essential to the implantation of the blastocyst is the process of decidualization where the endometrial stromal cells differentiate into secretory decidual cells to support embryo attachment and placenta formation.^125^^,^^126^ Unsuccessful decidual remodeling results in recurrent implantation failure and pregnancy loss.^127^ Using *Rb1cc1/FIP200* conditional KO in the uterus of mice and human endometrial stromal cells (hESC) in culture^67^ and a conditional *Atg16l1* KO female mice,^68^ fertility was reduced through a decrease in implantation rate, due to improper decidualization. Further, *ATG9A* regulates mitophagy to increase prolactin (PRL) secretion and induce decidualization in hESC,^128^ while KD of *ATG7* and *ATG5* hinders this process.^69^ A recent study in mice showed that *Becn1*-deficient uteri resulted in loss of endometrial progenitor stem cells, impacting proper uterine development.^70^ Altogether, autophagy is required for decidualization, a fundamental uterine cell differentiation process that assures implantation and placentation. There is still a need to understand how autophagy is precisely regulated in the various cells of the endometrium, how it supports stromal cell differentiation, how it participates in the menstrual cycle, why it is downregulated following implantation, and what role it has in many uterine diseases.^129^

## Placenta development and function

**Figure 3:**
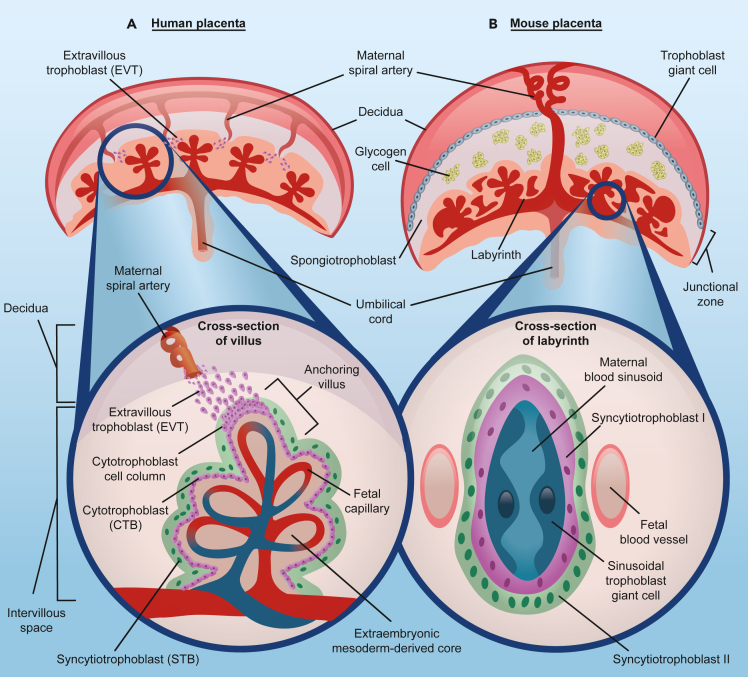
Structure of the fully developed human and mouse placentas (A) *Structure of the human placenta* - It is composed of three main cell types-extravillous trophoblast (EVT), cytotrophoblast (CTB), and syncytiotrophoblast (STB). The maternal decidua is the site where maternal circulation is established and where the placenta anchors to the uterine lining. The invasive EVTs penetrate the uterine wall to remodel the spiral arteries to ensure adequate blood supply to the growing fetus. Cross section of the human placental chorionic villi illustrates the cells in the fetal-maternal interface. The fetal blood vessels are surrounded by the layer of CTBs. Some CTBs detach from the basement membrane to form CTB cell columns, which contribute to the formation of the anchoring villi. The mononucleated CTBs in the villi fuse together to form the STB, a syncytium layer of nuclei and cytoplasmic content. This layer is in direct contact with maternal blood, secretes protein and hormones, and is involved in nutrient exchange with the underlying fetal circulation. (B) *Structure of the mouse placenta* - It is composed of three layers. 1) Maternal decidua which consists of invaded trophoblast giant cells that anchors the placenta to the uterus; 2) Junctional zone which is formed by glycogen cells and spongiotrophoblast. These cells control the metabolism and secretory functions of the placenta in the mouse; 3) Labyrinth zone is the region where fetal-maternal exchange occurs. The cross-section of the labyrinth illustrates the multiple cell layers between the maternal sinusoids and fetal blood vessels. Sinusoidal trophoblast giant cells line the maternal sinusoids, in direct contact with syncytiotrophoblast-I and -II cells. These cells form the barrier between maternal and fetal circulation, regulating nutrient exchange.

After blastocyst implantation in the uterine endometrium, trophectoderm cells aggressively proliferate, invade, and nest in the decidual bed. Once anchored, the placenta is formed as the physical and biochemical interface between the host and the developing embryo (Figure 3). Placental mammals have evolved species-specific structures and strategies to form a functional placenta depending on the developing environment and the needs of the embryo.^130^^,^^131^ In humans, the proliferative epithelial stem trophoblasts, the cytotrophoblasts (CTBs), fuse to form the syncytiotrophoblast (STB), and later the characteristic villous structure for gas and nutrient exchange with the maternal interface. Additionally, CTBs differentiate into extravillous trophoblasts (EVTs), which migrate and invade the maternal uterine spiral arteries to control maternal blood flow during pregnancy^132^ (Figure 3A). In mice, embryonic mesoderm differentiates to vascularize the yolk sac that is eventually replaced by the chorioallantoic placenta (Figure 3B).^133^

Initial human placenta formation triggers a persistent and severe hypoxic environment driven by trophoblasts plugging the uterine spiral arteries that last up to 10 weeks.^134^ After the first trimester, the plug dissolves, and EVTs replace endothelial cells remodeling maternal spiral arteries to gain access to maternal blood causing oxygen levels to return to normoxia.^132^^,^^135^^,^^136^ The low oxygen tension limits metabolism and reactive oxygen species (ROS) production in the embryonic compartment^137^ but it also triggers trophoblast differentiation and proliferation. Autophagy activation is observed in EVTs as an adaptation to hypoxia to maintain cellular homeostasis via a reduction in mTOR activity.^71^^,^^138^ Hypoxia inducible factor 1 subunit alpha (HIF1α), a master transcriptional regulator of the adaptive response to hypoxia, also regulates the invasion of trophoblast cells by increasing autophagy.^139^ Autophagy-deficient EVT-derived placental cells fail to show invasion, with decreased adenosine triphosphate (ATP) content following hypoxic conditions.^71^^,^^72^^,^^73^ Once EVTs adopt endothelial characteristics and remodel the spiral arteries, metabolic adaptations occur under these aerobic conditions in the placenta. Efficient remodeling is necessary to prevent trophoblast damage due to ischemia-reoxygenation injury and placental oxidative stress, including ER stress and inflammatory stress.^140^ Excessive oxidative stress results in trophoblast senescence by autophagy inhibition and decreased autophagic flux in a p53/mTOR-dependent fashion.^141^ Yet, oxidative stress was also shown to induce autophagy within trophoblasts and endothelial cells, reducing trophoblast invasion and placental vasculature.^142^ These contradictory observations require further dissection of autophagy responses to oxidative stress during placenta development.

In both mice and humans, STBs are responsible for exchanging essential molecules between fetal and maternal blood, among other secretory functions.^143^ This multinucleated STB (two contiguous layers in the mouse) is formed by cell-cell fusion of precursor cells by a process known as syncytialization.^144^ Failure of cell-cell fusion is implicated in placental pathology. The exact mechanism is still not clear; however, several players have been identified including fusogenic proteins (syncytins), various cytoskeleton elements, galectins, and phosphatidylserine exposure.^145^^,^^146^^,^^147^ Autophagy is considered a contributing mechanism to trophoblast syncytialization.^148^ Placental CTBs utilize autophagy for energy replenishment and cytoplasm remodeling when experiencing strong stimuli for syncytialization.^73^^,^^75^ Blocking autophagy flux by using bafilomycin A1 suppresses CTB fusion as shown by a decrease in human chorionic gonadotropin (hCG) secretion from STBs.^74^ On the other hand, activating autophagy using rapamycin results in excessive syncytialization in a pregnant mouse model.^75^ A recent study examined the expression of autophagy markers during a 96-hour time course of syncytialization in villous CTB cells derived from the human term placenta.^76^ They observed a gradual increase in the formation of acidic compartments and the autophagy marker LC3B. By manipulating the levels of BECN1, authors found that the inhibition or overactivation of autophagy led to a decrease in the cell fusion index, indicating the importance of tightly regulated autophagy during syncytialization.

Lysosomes are key organelles that respond to nutrients and cellular stress by activating multiple signaling events to recover from cellular damage and have an essential role in the late stages of autophagy^149^ (Figure 1). Although very low levels of the transcription factor EB (TFEB), a master transcriptional regulator of lysosomal biogenesis,^150^ are found in embryos, its expression is very evident in the trophectoderm and placenta,^151^ specifically in the labyrinthine trophoblast cells of mice.^77^ Homozygous mice carrying a germline mutation in *Tfeb* die between 9.5 and 10.5 days of embryonic development as they fail to express vascular endothelial growth factor (VEGF). Therefore, these mice lose normal placental vascularization.^77^ *In vitro* trophoblasts isolated from third trimester placenta and cultured under hypoxia showed a decrease in the levels of TFEB and several lysosomal proteins such as lysosomal associated membrane protein 1 and 2 (LAMP1, LAMP2), and cathepsin D (CTSD), with the concomitant impairment of autophagic flux, as observed by a decrease in the number of autophagic vacuoles.^78^ This was further verified in TCL-1 cells (third trimester EVT cell line) and an autophagy-deficient EVT trophoblast cell line (HchEpClb-ATG4B^C74A^), pointing to the role of autophagy in TFEB localization and lysosomal function in the placenta.^78^ Remarkably, three recent studies have found TFEB as a direct transcriptional regulator of trophoblast fusion proteins such as syncytins, independently from its canonical lysosomal targets,^152^^,^^153^^,^^154^ highlighting a novel placenta-specific dual gene program of TFEB and a better explanation for murine *Tfeb* KO vascular phenotypes.

In addition to its role in maintaining cellular homeostasis and trophoblast differentiation, autophagy plays a crucial role in safeguarding the feto-placenta unit against pathogens. Selective autophagy of pathogens is involved in recognizing and removal of invading intracellular bacteria and viruses.^155^ Placental explants deficient in *ATG16L1* exhibit heightened susceptibility to infections caused by pathogenic *E. coli*.^79^ On the other hand, the infection of human trophoblast cells and mouse placentas by Zika virus (ZIKV) induced the activation of autophagy.^80^ In mouse models with *Atg16l1* gene deficiency, ZIKV infection is reduced in both placentas and fetuses, resulting in improved fetal outcomes.^80^ However, in another study using human and non-human primate placenta-derived STBs, authors observed a decrease in expression of *ATG5* and *SQSTM1* and a decrease in protein levels of LC3B and SQSTM1 in cells with active ZIKV replication.^81^ This highlights the differential ability of various pathogens to exploit the autophagy mechanism for their intracellular survival in different species, underscoring the importance of autophagy in favoring or preventing the vertical transmission of infections from the placenta to the fetus.

## Parturition

At the end of every pregnancy, the process of labor begins and includes several stages: early or latent (body preparation), active (delivery of the fetus and expulsion of the placenta), and fetal-to-neonate transition. These processes are propelled by hormones and require very intense physiological responses for both the host and the fetus. Autophagy, despite being studied for its role in earlier stages of pregnancy, has not been extensively studied during delivery given practical restrictions. One study in humans points to more LC3-II in the placentas of non-labor cesarean birth compared to labor deliveries.^156^ Similarly, fetal membranes obtained after spontaneous term labor showed lower expression of the autophagy proteins BECN1, ATG3, ATG5, ATG7, ATG12, and ATG16L1 compared with term non-labored deliveries.^157^ However, these phenotypes could be due to patients fasting prior to the surgery and/or the use of anesthetics during cesarean section.^158^ No changes in the expression of several autophagy markers were observed in the placentas from the spontaneous onset of labor or pharmacologically induced parturition.^159^ *ATG16L1* affects the timing of delivery during labor induction.^160^ Single nucleotide polymorphisms in *ATG16L1* in the maternal genetic makeup were also found to be associated with pre-term delivery when hCG levels during the first 12 weeks were elevated.^161^

Autophagy is activated in the human uterine myometrium during labor and might play an important role in maintaining uterine contraction function.^162^ RNA-seq data analysis in the myometrium cells during labor showed significant enrichment in protein ubiquitination pathways, which is correlated with labor duration. Expression of genes involved in autophagy and vesicle transport proteins were also upregulated during the active phase of labor onset, normally lower in the latent phase of labor.^163^

Early neonatal survival depends on autophagy. Two fundamental studies in *Atg5* KO^87^ and Ras related GTP binding A (small GTPase that regulates mTORC1 activity) *Rraga*^Q66L^ mutant mice^88^ demonstrated that the interruption of sudden nutrient supply through the placenta during delivery triggers AMPK- and mTORC1-dependent autophagy in the neonate to maintain an adequate pool of nutrients until lactation takes over. Autophagy is actively induced right after birth in almost all the tissues^87^ and in line with this observation, conventional *Atg3*, *Atg5*, *Atg7*, *Atg9a*, *Atg12*, and *Atg16l1* null mice present fetal growth restriction, decreased plasma amino acid levels, and lead to pre-natal or neonatal lethality within one day of birth.^87^^,^^89^^,^^90^^,^^91^^,^^92^^,^^93^^,^^94^

In conclusion, the process of labor involves multiple stages and intense physiological responses in both the host and fetus. Autophagy, although less studied during delivery, has been found to have potential involvement and novel experimental approaches are needed to fully understand the mechanisms and significance of autophagy in the context of labor and delivery.

## Models to study human pregnancy in a dish

This section summarizes the development of different *in vitro* tools focusing primarily on models to mimic the human maternal-fetal interface. The development of models for gonads, germ cells, and embryos (or embryoids) is covered in several other specialized articles.^164^^,^^165^^,^^166^^,^^167^^,^^168^^,^^169^ Similarly, trophoblast monolayer cultures have been employed for over 50 years, providing valuable insights into placental barrier function, trophoblast invasion, toxicology, and disease modeling *in vitro*, are extensively reviewed elsewhere^170^ and therefore we will focus on recent innovations in cell lines and organoid models for studying human placenta physiology and pathology.

### *In vitro* models to study autophagy in the placenta

Recently, a breakthrough in the derivation of human trophoblast stem cells (TSCs) has been reported.^171^ Okae and colleagues demonstrated the derivation of TSCs from CTBs isolated from first-trimester human placental villi by manipulating signaling pathways (activation of Wnt and EGF pathways, inhibition of TGFB, and histone deacetylase-mediated inhibition of ROCK). These TSCs can be maintained in culture long-term (approximately 80 passages) and undergo directed differentiation into EVT- and STB-like cells. This study provides a detailed description of culture conditions and shows that the differentiated cell types closely resemble transcriptomes and epigenomes of *in vivo* human EVTs and STBs. The authors were unable to derive human TSCs from the term placenta, speculating that the CTBs that served as the origin for first trimester-derived lines are lost during the second trimester of pregnancy. This suggests the possibility of multiple CTB subpopulations throughout human pregnancy, although this has not been investigated yet. However, several years later, another group reported isolation and prolonged culture of trophoblasts from term placenta – a tissue that is easier to obtain compared to first-trimester placenta.^172^ Both first trimester- and term-derived trophoblast cultures as well as stem-cells-derived methods^173^^,^^174^^,^^175^ are great tools to investigate autophagy mechanisms as they have laid the groundwork for the establishment of 3D models to study placenta development.

Shortly after the emergence of methods to isolate and culture human trophoblasts, two groups generated trophoblast organoid models. In the first model, Haider et al. used purified first-trimester CTBs to establish a human placental organoid culture system.^176^ Similar to the Okae 2D culture, these organoids spontaneously differentiate into STBs. However, when self-renewal factors are removed from the culture, CTBs begin to express NOTCH1 (known EVT progenitor marker) and eventually form adjacent, distally located EVTs. In a parallel experiment, Turco et al. also successfully generated placental organoid cultures that follow similar developmental characteristics under specific conditions.^177^ Both methods rely on access to first-trimester tissue. However, more recent studies offered new alternative sources of these cells to generate placenta organoids: term placentas^178^ and stem cells.^179^^,^^180^^,^^181^

The current limitation of original 3D models is the inverted architecture of organoids – the STB lacks an outer apical membrane exposed to the culture medium and therefore does not recapitulate the *in vivo* shedding, making it difficult to study the maternal-fetal exchange of nutrients and gases. Interestingly, placental organoids are among many other *in vitro* tissue systems that face this challenge. Scientists are currently pursuing a workaround for this problem and a recent work shows a promising solution by culturing the organoid first in extracellular matrix domes and then resuspending it for proper cellular orientation.^182^ Other approaches implement several “organ-on-a-chip” systems, commonly referred to as “placenta-on-a-chip.”^183^ Although these systems have not yet solved the impediment to study syncytialization in 3D, they do provide new tools to investigate EVT invasion. Multiple labs have recently demonstrated new capabilities of reconstructing the 3D structure of the maternal-fetal interface to study *in vivo*-like invasion of EVTs into the host uterus. In one study,^184^ through testing multiple variations of cellular environments, authors employ such a system to show the importance of decidualized stromal cells in regulating EVT invasion and provide new evidence for the role that pre-implantation maternal immune cells play in this process. Another study focused on examining the engineering aspects of the model and its potential applications.^185^ When combined with fluorescent cell tagging and flow cytometry, the platform allows the collection of the invasive cells,^185^ opening doors for researchers to manipulate the autophagic network in ways that have previously been impossible in live animal models. The effects of these manipulations can be further investigated by biochemical profiling control and experimental trophoblast cells.

### *In vitro* models to study endometrium-placenta interactions

Perhaps a more detailed investigation of the autophagy network can be achieved by modeling trophoblast-endometrium interactions in 3D. To date, several *in vitro* systems have been designed to model such interactions.^186^ The first 3D models began to emerge in the mid 1980s and have become an invaluable tool to study mechanisms of embryo implantation, drug development, and disease modeling. These models often involve co-culturing primary cells and establishing cell lines in the presence of collagen, agarose gels, or Matrigel to recreate physiologic architecture. Supplementation of culture media with estrogen and progesterone is used to mimic physiological conditions.^187^^,^^188^^,^^189^^,^^190^ Models of endometrium have been used to investigate the impact of pharmaceutical compounds on endometrial receptivity^191^^,^^192^ and can be similarly employed to study the mechanism of autophagy. An alternative to cell-based 3D cultures to model endometrium is an *ex vivo* tissue approach.^193^ This model uses human endometrial tissue explants and incorporates an air-liquid interface into a 3D matrix scaffold using a type I collagen gel. Furthermore, a combined 2D/3D model to study implantation has been reported.^194^ In this model, three cell lines (HEC-1, Ishikawa, and RL95-2) were seeded in Matrigel until solidified (3D) and then overlaid with the same cell type (2D). Finally, a non-invasive method for the isolation of endometrial epithelial organoids and stromal cells from menstrual fluid has also been generated.^195^ Similar approaches can be combined in the future to fully understand human placental and endometrium interactions at the different stages of pregnancy.

The *in vitro* models described above provide a valuable platform for monitoring autophagy regulation, where it is difficult to obtain primary human tissue. Additionally, these novel cellular systems are more amenable to interrogation using classical tools and assessments^196^^,^^197^^,^^198^^,^^199^ such as autophagy flux assays, electron microscopy, and live-cell imaging of fluorescent reporters. Moreover, unbiased functional screens such as CRISPR or siRNA can be employed to identify novel cell-specific regulators of autophagy, broadening our understanding of its regulation, such as recently proven useful using human trophoblast stem cells.^200^^,^^201^ The novel hits from those screens can be further validated through orthogonal assays both *in vitro* and *in vivo*. Taking the advantage of these modern systems can help us better understand the role of autophagy in various aspects of reproductive biology.

## Autophagy in human pregnancy disorders

Autophagy has been implicated in the most prevalent human pregnancy disorders. Here we summarize such evidence from both mouse and human studies.

### Placenta accreta spectrum

The placenta accreta spectrum pathology is characterized by abnormal trophoblast invasion, permitting the placenta to deeply invade into the myometrium.^202^ This process is often compared to the behavior of invasive cancerous cells which involves epithelial-to-mesenchymal transition (EMT). Dysfunction of trophoblast cells or defect in endometrial-myometrial interface from failure in proper decidualization, results in placenta accreta pathology. Limited research points toward potential disease association with autophagy. Microarray analysis on normal and placenta accreta placentas revealed transcriptomic differences in genes related to autophagy.^203^ Chen et al. observed high expression levels of HIF1α, BECN1, SQSTM1, and LC3B in trophoblasts from pathologic placentas.^204^ Whether autophagy upregulation is hypoxia-driven or the cause for the invasive trophoblast phenotype in the placenta was not addressed. Evidently, more studies on these placental invasive phenotypes are required to understand the role, if any, of autophagy in both the pathogenesis and progression of placenta accreta pathology.

### Preterm birth

Preterm birth (PTB) refers to births occurring before 37 weeks of pregnancy, characterized by the weakening of fetal membranes. The majority of PTBs do not have clearly defined risk factors.^205^ Altered steroid levels during pregnancy, oxidative stress, and infection-driven inflammation mark major risks for PTB.^206^^,^^207^ These stressors are associated with aberrant EMT in cells of the fetal membranes leading to pregnancy complications associated with PTB.^208^ In a recent study using transcriptome analysis of amniochorion membranes from preterm pregnancies revealed the dysregulation of autophagy and EMT in fetal membranes.^209^ Brickle et al. utilized human placenta tissues obtained from spontaneous term and preterm labor and observed a decrease in protein expression of BECN1 and ATG7 in spontaneous preterm labor fetal membranes.^157^ A decrease in autophagy was further associated with high IL-1β levels and increased inflammasome activation, which contributed to spontaneous PTB.^157^ Infection-driven inflammatory conditions also affect autophagy flux, with a decrease in levels of ATG7, ATG16L1, and ATG4, and increased accumulation of LC3B.^79^^,^^207^ Studies with *Becn1* KO in precursor follicular granulosa murine cells show defects in progesterone production in the corpus luteum resulting in failure of decidualization and predisposition to preterm labor.^63^ Despite the largely unknown risk factors for PTB, autophagy appears to play a significant role, but its extent remains unclear.

### Intra-uterine growth restriction

In intra-uterine growth restriction (IUGR), the fetus fails to reach its full genetic growth potential resulting in pre- and postnatal complications. It is also one of the risk factors for preterm labor. Among various factors that lead to IUGR pathology, abnormal placenta development contributes as a major determinant.^210^ These placentas are smaller in size and have defective patterns of CTB invasion and proliferation, with the impaired remodeling of the maternal spiral arteries resulting in low levels of nutrient supply to the growing fetus. Marked changes in placenta autophagy have been observed in pregnancies resulting in IUGR. Placenta-specific *Atg7* KO mice showed a reduction in fetal growth, which was due to reduced placental weight.^82^ Interestingly, STB-specific *Atg7* KO inhibited fetal growth without affecting placental weight.^83^ Fetuses from KO mouse models of autophagy essential genes such as *Becn1,* *Atg3,* *Atg5,* *Atg7,* *Atg9a,* and *Atg16l1* showed an IUGR phenotype and died on day one after birth, thus implying the necessity of the autophagy process soon after birth.^79^^,^^87^^,^^90^^,^^91^^,^^92^^,^^95^ Remarkably, IUGR placentas showed an increase in the total number of autophagosomes in STBs.^211^ Further, primary CTBs from these placentas showed higher levels of ER stress, lower levels of mTOR phosphorylation, and increased expression of mTOR inhibitor TSC2 when subjected to oxygen-glucose deprivation (OGD) treatment.^96^ mTOR activity in fetal growth-restricted pregnancies is regulated by placental AKT and AMPK.^96^ However, these effects were not observed in *in vitro*-cultured CTB cells exposed to OGD. Loss of placental-specific mTOR function (mTOR-KO^placenta^) in a mouse model leads to reduced placental function and impaired metabolic health of the offspring.^97^ In conclusion, these phenotypes suggest that autophagy deficiency can cause the IUGR by impairing nutrient supply and placental function.

### Gestational diabetes mellitus

Gestational diabetes mellitus (GDM) is a type of diabetes that occurs during pregnancy, affecting both the host and the fetus. It alters fetal metabolism and placental uptake of nutrients, increasing the chances of hypertensive pregnancy disorders and risks to the fetus, including macrosomia and the development of diabetes and cardiovascular disease as an adult.^212^ Changes in nutrient availability are often sensed by cellular autophagy, which is associated with the development of insulin resistance in hosts during gestational diabetes.^213^ In GDM, fetal pancreatic beta-cells and placental STBs exhibit heightened LC3 and SQSTM1 expression, while placental BECN1 decreases, suggesting autophagy inhibition.^213^ Lower levels of ATG7 in the placenta are associated with risk factors of GDM in a population study of Chinese women.^214^ KD of *ATG5* in HTR8/SVneo cells exposed to high glucose levels showed decreased apoptosis and increased invasion. This suggests that a deficiency in autophagy may contribute to improved invasion capabilities.^84^ Increased phosphorylated mTOR and associated regulator molecules such as pAMPK were observed in the trophoblast villi of the placenta in GDM.^215^ Placentas from obese and GDM-affected pregnancies were shown to weigh heavier, which can lead to placental insufficiency and hypoxia resulting in poor feto-maternal exchange.^216^

### Preeclampsia

Preeclampsia (PE) is a hypertensive disorder of pregnancy, characterized by hypertension and proteinuria around 20 weeks of gestation. The exact mechanism is not understood; however, placental dysfunction is often associated with PE^217^ and autophagy may contribute to the development of this syndrome. Several studies have investigated the association between autophagy-related markers and hypertensive disorders in the placenta. Studies on human placentas showed that hypertensive disorders were associated with increased levels of LC3-II and decreased levels of SQSTM1.^218^^,^^219^^,^^220^^,^^221^ Enhanced expression of LC3 and BECN1 was found in placental STBs and vascular endothelial cells in patients with early-onset PE.^142^ In preeclamptic placentas, oxidative stress initiates an overproduction of ceramides.^85^ This, in turn, activates the expression of TFEB, leading to an increase in lysosome biogenesis and exocytosis. Accordingly, JEG-3 trophoblast cells exposed to external ceramides display heightened autophagy activation.^85^ These findings suggest that elevated autophagy may be a distinguishing feature of preeclamptic placentas. However, contrasting reports of autophagy impairment with higher SQSTM1 expression in PE-placentas have been described.^222^^,^^223^ In a placenta-specific *Atg7* KO mouse, which shows pregnancy-specific hypertension symptoms, an increase in SQSTM1 was observed in parietal trophoblast giant cells and the spongiotrophoblast layer of the placenta.^82^ These authors also observed lower levels of TFEB expression in both the labyrinth and junctional zone and high protein aggregates in the junctional zone.^78^ These findings suggest impaired autophagy and lysosomal function in PE placentas, contributing to protein aggregate accumulation. Although maternal blood pressure increased in the KO model, no proteinuria or IUGR was observed in the dams.^82^ Another autophagy regulator, PKCβ, a serine/threonine protein kinase, is downregulated in human PE placentas.^86^ Inhibition of PKCβ resulted in enhanced autophagic flux and an angiogenic imbalance in mouse placentas, which could be reversed by autophagic inhibition with 3-methyladenine (3-MA) treatment.^86^

Excessive protein aggregates were observed in trophoblast layers of villi and EVTs in placenta tissues from patients with PE.^78^ Lysosomes are key regulators for the clearance of protein aggregates. In the comparison of trophoblast cells from PE placentas to those of placentas from normal pregnancy and gestational age-matched deliveries, lysosomal markers LAMP1 and LAMP2 were notably absent and TFEB protein levels and nuclear translocation were reduced in trophoblast and anchoring EVTs.^78^ Studies from preeclamptic patients revealed the presence of aggregated transthyretin (TTR) in the placental tissue,^224^ while serum levels were reduced.^225^ In addition, aggregated TTR was also found in extracellular vesicles secreted from PE placenta tissue.^224^ TTR is a transporter of thyroxine and retinol that supplies thyroxine for the development of the fetal central nervous system.^226^ This protein was previously implicated in amyloid disease and associated aggregation in Alzheimer’s disease. It is also present in human trophoblastic cells and at the implantation site in mice.^227^ Moreover, studies have reported the presence of TTR protein aggregates in hypoxia/reoxygenation-exposed trophoblasts and preeclamptic placentas.^222^ Overexpression of human TTR in transgenic mice results in aggregated TTR in the placenta and PE-like features with an excessive unfolded protein response.^222^ Related to the presence of protein aggregates in pathological human placentas, fragments of amyloid precursor protein (APP) along with prototype APP processing enzymes α-secretase ADAM10, β-secretases BACE1 and BACE2, and the γ-secretase presenilin-1 were all upregulated in PE, which could be detected in urine samples.^228^ Accumulation of unfolded and misfolded proteins can activate ER stress-response pathways, leading to placental dysfunction. This stress contributes to early-onset but not late-onset PE.^229^ Further investigation is necessary to better understand the relationship between ER stress, autophagy, and placental dysfunction in PE.

### Spontaneous miscarriage

Spontaneous miscarriage (SM) is the loss of pregnancy before the 20th week of gestation. A number of factors such as poor decidualization, uterine abnormalities, endocrine dysfunctions, genetic defects, immunological factors, and environmental stress can be responsible for the etiology of SM.^230^ Autophagy can compensate for a variety of stressors during placenta development. Various studies have shown an increased expression of autophagy markers LC3, ATG5, and BECN1, along with decreased mitofusin-2 (MFN2), a protein involved in mitochondrial dynamics, in trophoblastic villi of the miscarriage placentas, indicating dysregulated autophagy in SM.^231^^,^^232^ Avagliano et al. demonstrated that STB from SM placentas exhibit increased LC3 levels as compared to normal placentas and a higher prevalence of autophagic structures by electron microscopy. However, additional experiments are warranted to understand the underlying cause of the high autophagy in SM.^159^ SM placentas also showed high levels of HIF-1α in villi and decidua, pointing to autophagy as a protector from hypoxia-induced apoptosis.^159^ Macrophage metabolite lysophosphatidic acid (LPA) can enhance the formation of autolysosomes, disruption of which was shown to induce SM by impairing trophoblast invasion and placenta development.^99^ Rapamycin may reduce the risk of miscarriage by enhancing endometrial and decidual macrophage autophagy which improved embryo absorption in SM mice models.^99^^,^^100^ As a result, autophagy activation might be a compensatory mechanism for mitigating SM-induced cellular stress.

## Conclusions

The environment has a significant effect on reproductive processes and plays a vital role in producing healthy offspring. Metabolic decisions are intricately linked to resource availability and environmental cues requiring a careful balance. When organisms face adverse conditions, autophagy becomes a key mechanism of cellular survival. Serving as a molecular gatekeeper, autophagy facilitates cellular function and development under stress by degrading and recycling cellular components, particularly during nutrient deprivation. Its tight regulation ensures the continuity of essential cellular functions, even amidst unfavorable external conditions. Our review shows that autophagy not only functions as a homeostatic process but may act as a dynamic regulator that senses and adapts to the shifting metabolic and environmental stressors at each step in reproduction. Mechanistically, autophagy can control the timing of cell differentiation, the amount of competent germ cells and fertilized embryos, the window and efficiency of implantation, the correct development of the placenta and its function while the fetus grows, and the expulsion of a healthy offspring, ready to survive outside the host. It is even required for cellular defense during pregnancy and immediately after delivery, controlling the maternal immune system from fetal rejection and excessive inflammation and protecting the new organism from infection. Autophagy appears to play a dual role: one that balances cellular demands with resource availability while also guiding complex signaling pathways that determine cellular fate. This layered control mechanism may represent an evolved strategy where autophagy ensures reproductive success by continuously monitoring and responding to the delicate interplay between metabolic shifts and cellular stress during early pregnancy.

**Figure 4:**
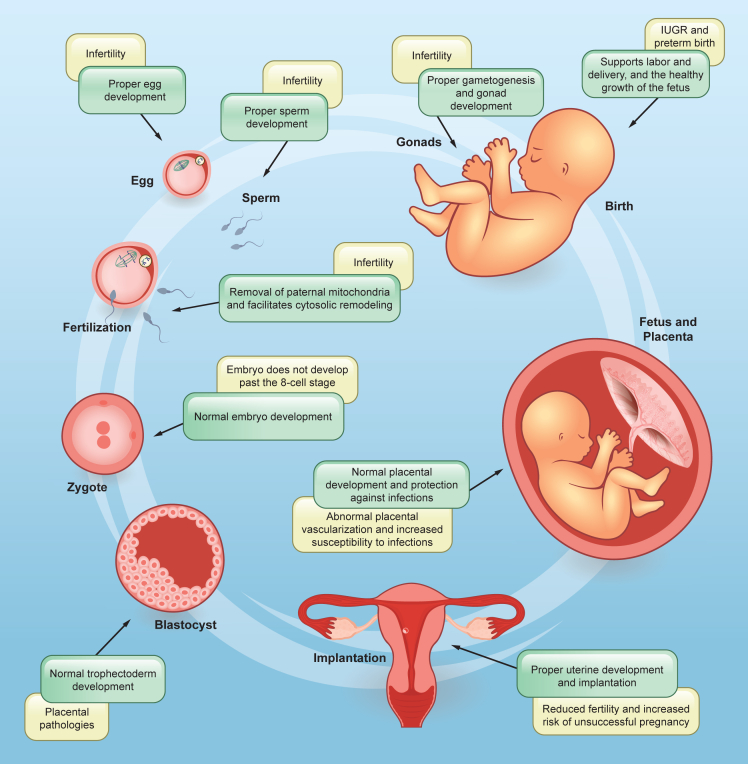
Key roles of autophagy in the different stages of pregnancy and associated pathologies The process of pregnancy requires strict mechanisms in order to ensure quality gametes for fertilization, successful implantation, formation of a healthy placenta and fetus, and delivery. Alterations in autophagy pathway genes are associated with various abnormalities observed at different stages of pregnancy.

Although such a role in cell survival seems to be so essential, our review shows that autophagy is largely unexplored and understudied in the field of reproduction (Figure 4). Here are some examples of research gaps that, when addressed, could have a significant impact on the field: 1) Whether research has been conducted using only a few classical ATG genes (*ATG5*, *ATG7,* and *BECN1*) or in a handful of specific cell types, the picture is far from complete. A combination of tools and models must be used to address autophagy systematically, taking into account the developmental timing and cell type of each phenotype, the role of early events (autophagosome formation and LC3 conjugation) as well as late players (lysosomes); 2) The lack of good models to study the different aspects of reproduction *in vitro* or the challenges to properly study early stages of implantation and placental and fetal development in humans make this particular field slower than others where autophagy has been explored in much more detail (e.g., neurodegeneration). Developing new *in vitro* models that ethically and practically allow researchers to study this phenomenon will certainly solve this dilemma. In the meantime, animal models remain an invaluable tool; 3) A significant role for reproductive hormones in regulating metabolism and autophagy in the gonads, as well as proper steroid formation, needs further exploration, given mouse models with clear infertility phenotypes; 4) Similarly, selective autophagy mechanisms and the different cargo receptors largely identified in the recent years have not been explored in the context of reproductive cells. Studies involving the silencing of such specific pathways may provide mechanistic insights into how autophagy of cytosolic content can cause some of the phenotypes described in this review; 5) *In vitro* studies using placental cell lines and *in vivo* studies using mouse models have demonstrated the involvement of autophagy in regulating placental development and function. However, most studies focus on specific time points or pathological conditions, providing limited insight into the dynamic changes in autophagy during the entire course of placental development. Research involving human placental samples from different gestational stages and other animal models, including non-human primates, is necessary to validate the findings observed in experimental models; and 6) There has been limited progress in understanding how autophagy (and its associated pathways) function during delivery, the most physiologically stressful event of a pregnancy. The use of animal models to enhance labor and delivery interrogation, combined with cellular approaches to study placenta-uterine communication, cervical remodeling, placental detachment, and uterine involution, presents promising research directions. Additionally, clinical and epidemiological studies that identify autophagy-specific disease patterns, risk factors, and causal relationships to be tested in the laboratory could further help bridge existing knowledge gaps.

It is still unclear whether autophagy plays a role in pathogenesis or is a potential target for several major pregnancy disorders. The reason for this is that autophagy appears to play a dual role, a characteristic that has long been observed in cancer.^233^^,^^234^^,^^235^ In conditions such as placenta accreta spectrum and preterm birth, it may contribute to the pathology, while in others, it can be a response to cellular stress and damage caused by other mechanisms failing. The complexity of interpretation is a result of the intricate nature of each disorder: its manifestation and degree depend on a number of variables, including timing, specific cellular failure or environmental triggers, and the inter-organ or systemic response to the failure. It may be possible to begin studying autophagy’s role in specific diseases by mechanistically examining the pathogenesis of the disease, using animal and human cell models that use conditional KO lines, autophagy reporters, and activators and inhibitors to test different stressors.^197^^,^^198^^,^^199^ Different cells control autophagy levels under different stress conditions. The placenta, for example, has evolved to proliferate and differentiate under hypoxic conditions where other cells may struggle. Recent studies have demonstrated the complexity of autophagy regulation, and its connection to cell differentiation, intracellular pathways, and apoptosis, highlighting the need to study this pathway not in isolation, but as a whole cell biology phenomenon that spans a cell’s entire life cycle. The methods for evaluating autophagy, or the way samples are handled, can significantly impact the consistency of data. Future studies should focus on large scale studies and well-designed protocols to address the challenging questions in the field.

While many questions remain unaddressed, there are promising efforts being made in this field to demystify the role of autophagy in pregnancy-related diseases. The emergence of several *in vitro* organoid techniques, the improvement of cell lines and models to mimic human reproductive cells, the advancement of non-invasive techniques for monitoring pregnancy, and the invaluable resource that several animal models continue to provide for scientists make this field fertile soil for more discoveries and innovative approaches to therapies and diagnostic tools that could revolutionize the future of the reproductive biology field. Although numerous clinical trials are actively investigating various drug targets within the autophagy pathway for various diseases,^236^ the potential to apply this approach within the context of pregnancy, reproductive development, and related disorders remains largely unexplored, with a few exceptions.^129^ Thus, understanding the importance of these crucial phases in human life provides an opportunity to dive deeper into autophagy’s involvement in regulating these important developmental stages which may unlock new insights for future clinical trials and prevention strategies. The world will keep changing and understanding how the cell adapts to each new environment, no matter the stage in the reproductive cycle, could be the solution to the next generation’s problems.

## Acknowledgments

We thank members of the Guardia’s lab and Drs. Carmen Williams and Martin Estermann for helpful discussions, and Paul Windsor from the NIEHS Office of Communications and Public Liaison for assistance with figures. As space limitations and our focus on the most recent research in the field prevented us from including all important studies, we apologize for any omissions. We thank NIH Fellows Editorial Board for reviewing the article. This work was supported by the Intramural Program of NIEHS (ZIA ES103370-01).

## Author contributions

Conceptualization: A.S. and C.M.G.; writing – original draft: A.S., M.L.P, O.K, E.P.-B., and C.M.G.; writing – review and editing: A.S., M.L.P, and C.M.G.; visualization: M.L.P. and C.M.G.; project administration and supervision: C.M.G.; funding acquisition: C.M.G. All authors have read and agreed to the published version of the article.

## Declaration of interests

All authors declare no competing interests.
